# Artificial intelligence in academic writing and clinical pharmacy education: consequences and opportunities

**DOI:** 10.1007/s11096-024-01705-1

**Published:** 2024-03-12

**Authors:** Anita Elaine Weidmann

**Affiliations:** Department of Clinical Pharmacy, Institute of Pharmacy, Innsbruck University, Innrain 80, 6020 Innsbruck, Austria

**Keywords:** Academic, Artificial intelligence, Clinical pharmacy, Education pharmacy, Intelligence machine

## Abstract

The current academic debate on the use of artificial intelligence (AI) in research and teaching has been ongoing since the launch of ChatGPT in November 2022. It mainly focuses on ethical considerations, academic integrity, authorship and the need for new legal frameworks. Time efficiencies may allow for more critical thinking, while ease of pattern recognition across large amounts of data may promote drug discovery, better clinical decision making and guideline development with resultant consequences for patient safety. AI is also prompting a re-evaluation of the nature of learning and the purpose of education worldwide. It challenges traditional pedagogies, forcing a shift from rote learning to more critical, analytical, and creative thinking skills. Despite this opportunity to re-think education concepts for pharmacy curricula several universities around the world have banned its use. This commentary summarizes the existing debate and identifies the consequences and opportunities for clinical pharmacy research and education.

## Introduction

Few topics have captured the news headlines more than the launch and rapid development of artificial intelligence (AI) models for public use in 2023 [1]. In particular the debate over its use in academic writing and publishing at university and within the scientific community has been ongoing encompassing various ethical, practical, and philosophical dimensions [2–4]. The approach to the use of these models in student assignments varies significantly across universities worldwide, with some institutions opting for bans, others for integration and many for a more nuanced, case-by-case approach [5, 6]. This commentary aims to summarize the existing debate and identify the consequences and opportunities for clinical pharmacy research and education.

### The role of artificial intelligence in academic writing

The release of ChatGPT (Open AI) in November 2022 marked the proverbial “tip of the iceberg” when it comes to generative AI models available to support the academic research and writing process. There are a seemingly endless number of products available that use AI functions from the conception of research questions (e.g. Elicit AI), identifying appropriate scientific literature databases (e.g. Search Smart), conducting efficient literature reviews & analysis (e.g. Litmaps, Consensus, Connected Paper, ResearchRabbit, Scite, OpenRead), data interpretation & synthesis (e.g. ChatGPT4, ResearchGPT, Lateral), as well as structuring and writing academic assignments, scientific journal publications and funding grant applications (e.g. Jenni.ai, Quillbot) [not an exhaustive list]. While AI tools seem to speed up the efficiency of several processes; perform basic and more complex analysis of data (including statistical analysis); identify patterns and data trends, draw inferences facilitating the understanding of complex concepts and provide contextual information, it cannot complete intellectual reasoning, application and integration of knowledge across complex problems or display genuine creativity or develop novel theories [7, 8]. Their value depends upon the scale and range of sources used to enable them. These are not usually disclosed in detail and vary greatly from one tool to another as do the types of functions that have been programmed into them making them more or less valuable.

The ongoing debate within the scientific and academic community centres mainly around ethical considerations of academic integrity such as plagiarism. Critics argue that the use of AI in academic writing undermines the authenticity of scholarly work, potentially leading to plagiarism or the erosion of original thought and critical thinking skills [9]. Claiming authorship for AI generated content is also a widely debated topic as, legally speaking, artificial intelligence models cannot be responsible for the content they create [10]. The user of these models must take full responsibility, but how can they when it is not clear how the information is generated and what it is based on? The extent of human contribution in AI-assisted writing becomes complex and highlights the need for the legal frameworks around intellectual property (IP) to be adapted to allow the definition and protection of IP in this new context [11]. Considering the lack of user insight on how these models derive their output, significant concerns exist around objectivity, biases and fairness. There is an inherent risk of diminishing the quality of the academic work and oversimplification of nuanced academic arguments, resulting in the loss of innovation and novel critical thought [12].

In contrast, AI tools have the potential to drive the move towards the free and open availability of research papers, democratizing research and academic resources thereby levelling the “playing field” for all [13]. The time freed up by streamlining several tasks within the research process affords the researcher more time for critical thinking, nuanced analysis and intellectual processing [14]. Pattern recognition across vast amounts of data may also lead to new insights in drug discovery and development, predicting trends in pharmacy practice, promoting better clinical decision making and guideline development with potentially beneficial consequences for patient safety and cost to the healthcare system [15].

### The role of artificial intelligence in clinical pharmacy education

Around the world institutions are exploring how AI can be integrated into curricula in a way that enhances learning outcomes while maintaining academic rigor [16]. This integration of AI in teaching and curricula is prompting a re-evaluation of the nature of knowledge, learning and the purpose of education worldwide [13, 17]. It challenges traditional pedagogies, forcing a shift from rote learning to more critical, analytical, and creative thinking skills. The International Pharmaceutical Federation (FIP) believes that “*pharmacy education should take a needs-based, outcome focused approach*…*to allow for innovations and developments*” [18]. There is no doubt that the use of artificial intelligence models in student assignments, teaching, pharmacy curricula and practice skill-based education qualifies as such an innovation.

In a recent series of world café events, conducted with undergraduate pharmacy students at the University of Innsbruck, students voiced their clear expectation that the ethical and practical use of AI tools should be taught as part of the pharmacy undergraduate curriculum [unpublished data], presumably reflecting the views of many pharmacy students around the globe. Students even considered the idea that lectures and face-to-face knowledge transfer can be replaced by methods such as AI assisted learning in a flipped classrom style pedagogical format, with the time spent at university focused on the development of practice skills, application of knowledge in experiential settings and the development of critical analytical thinking skills [19].

This skills-based learning approach for pharmacists is nothing new and has been an integral part of accreditation standards for pharmacy education (both under- and postgraduate) around the world for the past two decades [20, 21]. A study from 2020 which compared the initial pharmacy education curricula from 16 countries and territories around the world however showed, that the content and emphasis of curricula are still very disparate and that accreditation standards are not present in all countries [22]. Curricula are often “*fragmented, outdated and static”* with vast differences in proportion of time dedicated between chemistry, physical science and practice [23]. Maybe AI will provide the necessary impetus for all pharmacy curricula to rethink their delivery and opt for a more skill-based learning and teaching approach. These could include more peer, collaborative and team-based learning opportunities, experiential learning and entrustable professional activities, interdisciplinary education, and reflective practice to self-assess understanding and knowledge [24–27]. More varied assessment formats such as open-book online exams using assertion-reason questions, objective structured clinical examinations including simulated/real interactions as well as practice portfolio style assessments should replace recall based written and verbal assessments. AI also offers opportunities to re-think research skills, allowing more data driven analytical teaching and a flipped approach to teaching the structure of scientific papers, good scientific writing practice and the development of a clear and concise writing style [28, 29]. In addition, AI may allow the development of personalized learning and intelligent tutoring systems which will allow tutors/ lecturers to better identify, and support struggling students and help students develop better time-management and planning skills [30]. Preparing students and adapting curricula for the implications of AI on clinical practice is imperative in this new era of pharmacy practice and education [31]. A recent paper by de Oliveira Santos Silva (2024) suggests a need for the training of “disruptive” educators capable of “*using teaching–learning methods adapted to the digital environment and educational processes suitable for stimulating the use of effective disruptive technologies.*” arguing that “*the pharmacy profession can no longer wait for the slow integration of digital technologies into pharmacy practice and education.*” [32]

So, if AI affords us the opportunity to re-think education concepts for pharmacy curricula why have several universities around the world banned its use? [33]. One can only surmise that it is a reaction to the speed at which AI technology is developing, to allow academic and scientific institutions time to better understand the ethical and legal implications as well as the practical impact these novel technologies have on current academic processes and allow the development of well thought out standards and regulations governing the use of AI in the academic settings.

## Conclusion

As AI technology continues to evolve, so will the debate. Continuous research into the implications, benefits and drawbacks of AI in academia is essential to inform new practices and policies moving clinical pharmacy practice, research and education along with this transformative technology. While there are several risks and grey areas, it may provide the necessary “disruptive” driving force to move pharmacy education forward in line with the professions ambition for clinical pharmacy practice.
